# The Chemical Deposition Method for the Decoration of Palladium Particles on Carbon Nanofibers with Rapid Conductivity Changes

**DOI:** 10.3390/nano6120226

**Published:** 2016-11-29

**Authors:** Hoik Lee, Duy-Nam Phan, Myungwoong Kim, Daewon Sohn, Seong-Geun Oh, Seong Hun Kim, Ick Soo Kim

**Affiliations:** 1 Nano Fusion Technology Research Group, Division of Frontier Fibers, Institute for Fiber Engineering (IFES), Interdisciplinary Cluster for Cutting Edge Research (ICCER), Shinshu University, Tokida 3-15-1, Ueda, Nagano 386-8567, Japan; hoik0822@gmail.com (H.L.); duynamphan@gmail.com (D.-N.P.); 2 Department of Chemistry, Inha University, Incheon 22212, Korea; mkim233@inha.ac.kr; 3 Department of Chemistry and Research Institute for Natural Sciences, Hanyang University, Seoul 04763, Korea; dsohn@hanyang.ac.kr; 4 Department of Chemical Engineering, Hanyang University, Seoul 04763, Korea; seongoh@hanyang.ac.kr; 5 Department of Organic and Nano Engineering, Hanyang University, Seoul 04763, Korea; kimsh@hanyang.ac.kr

**Keywords:** carbon nanofiber, hydrogen sensor, palladium-doped carbon nanofibers, conductivity change, volume change, chemical deposition

## Abstract

Palladium (Pd) metal is well-known for hydrogen sensing material due to its high sensitivity and selectivity toward hydrogen, and is able to detect hydrogen at near room temperature. In this work, palladium-doped carbon nanofibers (Pd/CNFs) were successfully produced in a facile manner via electrospinning. Well-organized and uniformly distributed Pd was observed in microscopic images of the resultant nanofibers. Hydrogen causes an increment in the volume of Pd due to the ability of hydrogen atoms to occupy the octahedral interstitial positions within its face centered cubic lattice structure, resulting in the resistance transition of Pd/CNFs. The resistance variation was around 400%, and it responded rapidly within 1 min, even in 5% hydrogen atmosphere conditions at room temperature. This fibrous hybrid material platform will open a new and practical route and stimulate further researches on the development of hydrogen sensing materials with rapid response, even to low concentrations of hydrogen in an atmosphere.

## 1. Introduction

The primary physical hazards associated with hydrogen gas are its flammability and potential for explosions; thus, numerous approaches are being investigated to develop hydrogen sensors [1]. The ability to selectively sense hydrogen is invaluable for a broad range of applications in the fields of environmental and civil infrastructures, climate and energy, health and safety, industry processing, hydrogen storage, and fuel cells [2,3]. Even though there have been significant efforts to enhance the sensitivity of hydrogen sensors, the increasing needs for better detection methods demand continuous efforts in developing a new material for hydrogen detection.

Palladium (Pd) is sometimes metaphorically called a “metal sponge” due to its excellent soaking up ability for hydrogen—“like a sponge soaks up water”. It has already been reported that Pd can absorb up to 900 times its own volume of hydrogen at room temperature and atmospheric pressure [4,5]. Pd is a widely used catalyst in the field of chemical sensors in light of its great selectivity towards hydrogen. The Pd/hydrogen system has been developed over several decades to address processes such as adsorption, desorption, and permeation [6]. Hydrogen is highly soluble in Pd, and it has a reversible process dependent on temperature and hydrogen partial pressure. 

The gas sensitivity and response speed are increased rapidly when the dimensions of sensing materials achieve one-dimensional geometry. Various 1D materials have been widely used to develop fast and efficient sensors such as carbon nanotube and Si nanowires [7,8]. One of the most attractive methods of fabricating 1D material is electrospinning. A variety of 1D nanofibers from a remarkable range of organic and inorganic materials have been prepared with the electrospinning technique successfully [9]. Electrospun nanofibers are featured with a very small diameter, an extremely long length, a large surface area per unit mass, and a small pore size, which provide a high surface-to-volume ratio [10]. The specific physical and chemical properties of the fabricated nanofibers make them versatile for various applications such as sensors, catalysis, optoelectronics, textiles, filters, fiber reinforcement, drug delivery, tissue engineering, and wound healing [11,12].

Carbon nanofibers (CNFs) have a nanostructure with remarkable electronic and mechanical properties. Its unique structure and outstanding properties have attracted scientific interest for decades. Potential applications of CNFs include polymer additives, gas storage materials, catalyst supports, and high power electrochemical capacitors [13,14,15]. Especially, carbon-based nanomaterials such as graphene, carbon nanotube, and CNFs have been widely exploited as catalyst substrates in hydrogen production due to their excellent resistance to corrosion, superior thermal stability, and mechanical strength [16,17].

Here, we report a simple fabrication method of one-dimensional palladium-doped nanofibers (Pd/CNFs) as a candidate for hydrogen sensing material. As mentioned, Pd is an excellent metal for the absorption and release of hydrogen in specific conditions, and nanofibrous architecture additionally offers unique characteristics such as a high surface area. The combination of these unique properties of Pd and nanofibrous structure provides great potential for hydrogen sensor applications. The synthesis of metal nanoparticles on CNFs has been reported in several publications [18,19]. Zhu et al. reported a linear decrease in resistivity in a Pd-decorated CNF web with an increase in the H_2_ volume fraction from 0 to 0.7 in a H_2_/N_2_ mixture gas, and showed good reversibility [18]. However, the Pd particles were deposited on the surface of carbon nanofibers by the supercritical CO_2_ method and followed by pyrolysis at 600 °C. The process was conducted in liquid CO_2_ at high pressure and followed by subsequent annealing at high temperature of 150 °C and 600 °C. Our chemical deposition process of simple dipping with a precursor solution having optimized pH could provide a less complicated way of decorating Pd particles on CNFs with rapid resistance changes compared with previous reports. You et al. demonstrated a Pd-decorated CNF system with the simple combination process of electrospinning and thermal treatment to investigate the electrocatalytic activities toward hydrogen peroxide and NADH [19]. In their method, a polyacrylonitrile/Pd(acetate)_2_ mixture solution was electrospun and followed by thermally treatment at 1100 °C. During the conversion of polyacrylonitrile nanofibers to CNFs, the Pd^2+^ ions in the nanofibers are reduced to Pd° and aggregated into Pd nanoparticles. Consequently, the total surface area of Pd particles is reduced due to the imbedding in CNTs than the particles located only on CNF surface. In this study, CNFs were produced from poly(acrylonitrile) (PAN) nanofibers through a stabilization and carbonization process [20], followed by a chemical deposition process conducted for the successful fabrication of Pd/CNFs. It was revealed that pH is critical to achieve specific and desirable Pd/CNFs; the optimized pH condition is pH 5 for fully doped Pd/CNFs. The chemical deposition method enables Pd particles to be easily decorated on CNFs and more effectively anchored on a CNF surface than previously mentioned reports. In addition, the Pd/CNFs showed a distinct electro-resistance change of around 500% after absorbing hydrogen, and revealed a rapid transition of resistance, even in an atmosphere of low hydrogen concentration.

## 2. Material and Methods

### 2.1. Materials

PAN (M_w_ 150,000 g/mol) and palladium chloride (PdCl_2_, >99.9%) were purchased from Sigma-Aldrich, Tokyo, Japan. *N*,*N*-dimethylformamide (DMF), ethanol, NaOH, and HCl were purchased from Wako Pure Chemical Industries, Osaka, Japan. DMF was used as solvent for fabricating PAN nanofibers by electrospinning. A mixture of PdCl_2_, ethanol, NaOH, and HCl was used as a dipping solution for Pd deposition. All reagents were used without further purification.

### 2.2. Characterizations

The surface structure and morphologies of the prepared nanofibers were studied with a scanning electron microscope (SEM; JSM-6010LA, JEOL, Tokyo, Japan) and a field emission scanning electron microscope (FE-SEM; S-5000, Hitach, Tokyo, Japan). PAN nanofibers (PAN NFs), stabilized nanofibers (ST-NFs) and CNFs were coated with Pt under a JFC-1600 fin coater (JEOL, Tokyo, Japan) for 60 s before taking microscope measurements. The average diameter was measured from the SEM micrographs using image analysis software (Image J, version 1.49, Bethesda, MD, USA). To obtain an average diameter and standard deviation, 50 points in a single SEM image were randomly selected. Elemental analysis was performed using a FE-SEM equipped with energy dispersive X-ray (EDX; S-5000, Tokyo, Japan) spectroscopy in order to investigate the Pd content of nanofibers. In order to characterize the chemical state of the Pd, X-ray photoelectron spectroscopy (XPS; Axis-ultra DLD, Shimadzu Co., Kyoto, Japan) analyses were performed. Resistivity measurements of CNF and Pd/CNF mats with and without exposure to hydrogen atmosphere were conducted with a four-point probe instrument. (Loresta GP MCP-T610, Mitsubisi chemical analytech, Kanagawa, Japan). 

### 2.3. CNF Fabrication Via Electrospinning

PAN NFs were fabricated via the electrospinning process. PAN (10 wt %), in DMF, was prepared at 60 °C through mechanical stirring for 2 h, and the temperature was then dropped to room temperature. An electrospinning apparatus installed with a high voltage power supply (Har-100*12, Matsusada Co., Tokyo, Japan), capable of generating voltages up to 100 kV, was used as the source of the electric field [21]. The high voltage is applied to the polymer solution that rests on a sharp conducting tip, resulting in molecular ionization or charge redistribution in the solution. When a sufficiently high voltage is applied to a droplet on the tip, the highly charged droplets have an electrostatic repulsion force that makes the solution erupt from the surface, which is known as the Taylor cone [22]. A copper wire connected to a positive electrode (anode) was attached to an ejection needle with an inner diameter of 0.6 mm, and a negative electrode (cathode) was linked to a metallic drum (collector). A voltage of 9 kV, a 15 cm tip-to-collector distance, and 0.4 mL/h flow rates (controlled via syringe pump) were employed. The electrospinning was carried out at room temperature and ~40% humidity. 

Before the carbonization of PAN NFs, it should be stabilized at a mild temperature to construct an infusible ladder form [20]. In the first step of producing CNFs, the prepared PAN NFs were stabilized in an air atmosphere at 300 °C for 1 h (heating rate was 1 °C/min) in an electric furnace (NHV-1515D, Motoyama, Co., Miyagi, Japan). To prevent the thermal shrinking of nanofibers, a PAN NF sheet was tied up with an alumina frame at the end of the sheet. ST-NFs were then carbonized as the temperature was increased to 900 °C at a heating rate of 5 °C/min in a nitrogen atmosphere. They were kept at the final temperature for 1 h. For the carbonization process, an electric heat-treating furnace (AMF-9P-III THV, Asahi Rika Seisakujo, Chiba, Japan) was used. Schematic illustration of fabricating CNFs and their SEM images are presented in Figure S1. 

### 2.4. The Pd Deposition Process on CNFs

A 5 mM PdCl_2_ solution in water/HCl was prepared with a 7:1 volume ratio for the chemical deposition of Pd ions on the surface of prepared CNFs. Ten milliliters of a prepared PdCl_2_ solution was mixed with 60 mL of water and 30 mL of ethanol (ethanol as reducing agent). The pH of the solution was adjusted by the addition of NaOH from 1 to 10 (the initial pH of solution without NaOH was 1). The prepared CNF sheet was immersed in the solution, and temperature was increased to 70 °C. The Pd deposition was carried out for 1, 6, and 12 h; they are denoted as 1 h·Pd/CNFs, 6 h·Pd/CNFs, and 12 h·Pd/CNFs, respectively. After the immersion process, the CNF sheets were washed with water and ethanol for removing residues.

### 2.5. Hydrogen Sensing Test

For hydrogen detection, a closed plastic chamber was connected to a gas flow system. Hydrogen (99.999%) and nitrogen (99.99%) were introduced to a gas mixer via a two-way valve using separate mass flow controllers. The test gases were allowed to flow through a pipe network with a diameter of 5 mm to a test chamber. The mixed gas was injected to a test chamber with a prepared NF. By monitoring the output voltage across the fixed NF length (1 cm), the resistance was measured in dry air and in a test gas. All absorption testing was conducted at room temperature. A simple illustration of the fabricated device for the hydrogen sensing test is depicted in Figure S2. 

## 3. Results and Discussion

The surface morphologies of fabricated the PAN NFs, ST-NFs, and CNFs were evaluated by SEM, which exhibit randomly oriented, bead-free, and smooth-surfaced nanofiber structures, and their fabrication process are depicted in Figure S1. Noticeably, it appears that they retain their fibrous morphology during the stabilization and carbonization process at high temperatures, resulting in a decrease in the fabricated NF diameter. Initially, the diameter of PAN NFs was reduced from 409 ± 62 nm to 363 ± 64 nm and 260 ± 43 nm, for the stabilized PAN NFs and CNFs, respectively. This diameter reduction may be attributed to the dehydrogenation, oxidation, and cyclization at higher temperatures [23]. Furthermore, thermal treatments were conducted under mechanical stress, such as tying up NF sheets with an alumina frame in the stabilization process in order to improve the cis-configuration of nitrile groups, thus improving the intramolecular cyclization. Since the stabilization and carbonization processes were carried under mechanical stress, the digital photographs in Figure S3 demonstrate wrinkle-free sheets. However, several pin holes were observed on the surface of the ST-NF and CNF as a result of thermal treatment. Fitzer et al. organized a mechanism and sequence of the carbonization process for PAN as well [23]. 

**Figure 1:**
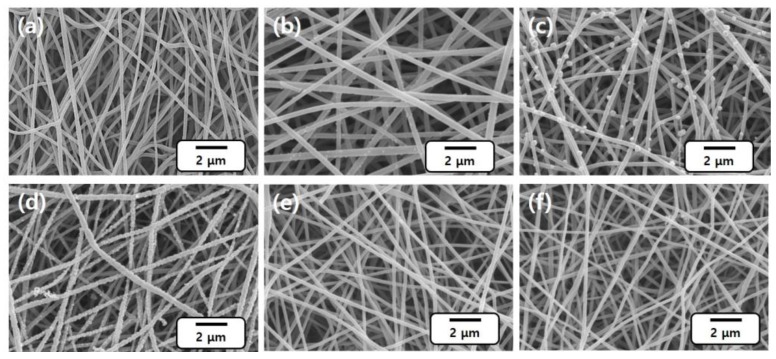
Morphology change of CNFs (carbon nanofibers) through chemical deposition in different pH solutions: (**a**) virgin CNFs; (**b**) pH 1; (**c**) pH 3; (**d**) pH 5; (**e**) pH 7; (**f**) pH 10.

The chemical deposition of Pd ions was performed on prepared CNFs by an immersion method. Pd ions on CNFs demonstrated different extraction behaviors with pH condition. Considering a previous report demonstrating a dependence of extraction rate on temperature (faster extraction rate at higher temperature) [24] and a boiling point of ethanol (78 °C), 70 °C was selected as the extraction temperature. To evaluate the effect of pH on the extraction, CNF sheets were immersed in PdCl_2_ solutions at different pH levels ranging from 1 to 10 for 1 h. The resulting morphology of the Pd/CNFs is shown in Figure 1. Figure 1b (solution pH 1) demonstrates fewer and small sized (around 70 nm) uplifts on the surface of CNFs. With pH increasing to 3, numerous and large-sized (100–300 nm) uplifts of Pd particles are evidenced (Figure 1c). For the pH 5 sample, the CNF surface is observed to be fully covered with Pd (Figure 1d). However, the sample for neutral (pH 7) and alkaline (pH 10) shows insignificant adsorption of the Pd uplifts (Figure 1e,f). The results indicate that weakly acidic pH is suitable for the extraction of Pd ions on CNFs, and the optimum pH value is pH 5 due to a comparatively higher adsorption of Pd extracts. Physical appearances of the PdCl_2_ solutions before and after adsorption of Pd, given in Figure S4, are in accordance with the results given in Figure 1, resulting in color changes of PdCl_2_ solutions. The yellow color originated from Pd ions; in a pH 1 solution, the sample keeps its yellow color after dipping the CNF, indicating that the Pd ions were not extracted. However, the yellow color turns pale yellow in a pH 3 solution and the solution finally becomes almost transparent above pH 5 after chemical deposition, indicating fully extracted Pd ions. Thus, it is strongly suggested that the pH of solutions is a significant parameter for the adsorption of Pd ions as the pH value affects the structural properties of adsorbents [25]. Above pH 3, the Pd hydroxide complexes such as Pd(OH)^+^, Pd(OH)_2_, or Pd(OH)_4_^2−^ start to form, resulting in extracting Pd on the CNF surface or precipitating in solution. As mentioned above in Figure 1e,f, the surface of the CNF sheets dipped in pH 7 and pH 10 solutions did not show any Pd deposition on the CNFs, which may be due to the extracted and precipitated Pd ions before their adsorption on the CNF surface due to its high extraction rate. It is notable that the interaction of Pd^2+^ ions with hydroxyl groups is more favored in an alkaline solution; thus, it can be easily reacted and extracted in a high pH condition. Based on these outcomes, pH 5 for the chemical deposition was set as an optimum condition, and the rest of the experiments were conducted at this pH. 

**Figure 2:**
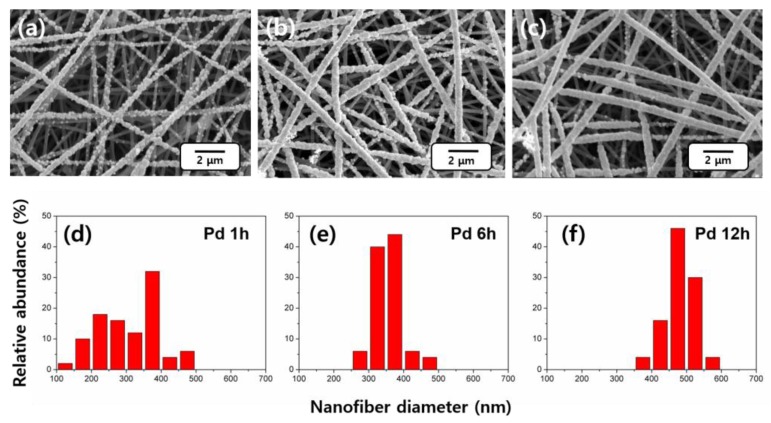
Scanning electron microscope (SEM) images of chemically deposited Pd ions on CNFs with different times: (**a**) 1 h; (**b**) 6 h; (**c**) 12 h. The images are accompanied by the corresponding diameter distributions in (**d**–**f**).

The results of the deposition of Pd^2+^ ions with different times were observed in SEM images, which are presented in Figure 2. The chemical depositions were carried out for 1 h, 6 h, and 12 h respectively, confirming that time governs the growth of the extracted amount of Pd. The surfaces of the CNFs are covered with Pd uplifts, which grow in number and size as the chemical deposition time increases. As a result, the CNF surfaces are largely covered with Pd uplifts (Figure 2b). Upon the further increase in time to 12 h, the size increase of the Pd uplift is clearly confirmed (Figure 2c). The average diameters of the 1 h, 6 h, and 12 h samples were 310 ± 84 nm, 370 ± 47 nm, and 498 ± 38 nm, respectively. Additionally, the standard deviation of the diameter decreases with increasing deposition time. This result indicates that Pd ions are deposited in the uncovered space on the CNF surface, leading to growth accordingly and hence a smoother coating with extended time. This in turn produces a more even coating of Pd and a narrow diameter distribution with decreased standard deviation (Figure 2d–f).

**Figure 3:**
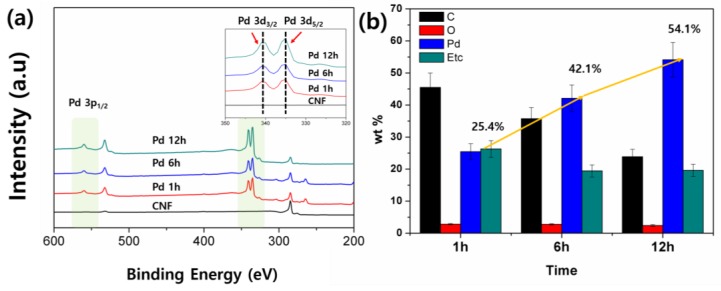
Probing Pd decoration process on CNFs. (**a**) The X-ray photoelectron spectroscopy (XPS) spectra of CNFs and Pd/CNFs with different deposition times. Inserted spectra magnified in Pd 3d peaks of Pd/CNFs; (**b**) elemental analysis conducted by energy dispersive X-ray (EDX) shows an increment in Pd content in CNFs as deposition time increases.

To support the proposed extraction mechanism on the CNFs, XPS and EDX spectroscopy were conducted. The EDX results presented in Figure 3b and Figure S5 confirm the increment of Pd on the CNFs. The results further justify the presence and subsequent increase in Pd content with chemical deposition time such that 25.4 wt % of Pd for 1 h increases to 54 wt % for 12 h. The others indicate the signals from the residual chemicals and the substrate. The XPS spectrum (Figure 3a), besides the C 1 s peak at 285.0 eV and O 1 s peak at 532 eV [26], shows Pd 3d_5/2_ and Pd 3d_3/2_ peaks at 335.1 eV and 340.7 eV, respectively, in good agreement with the previous reports of bulk Pd(0) [27,28]. The peak intensity increases with the increase in deposition time at the Pd region is in accordance with the SEM and EDX results. 

**Figure 4:**
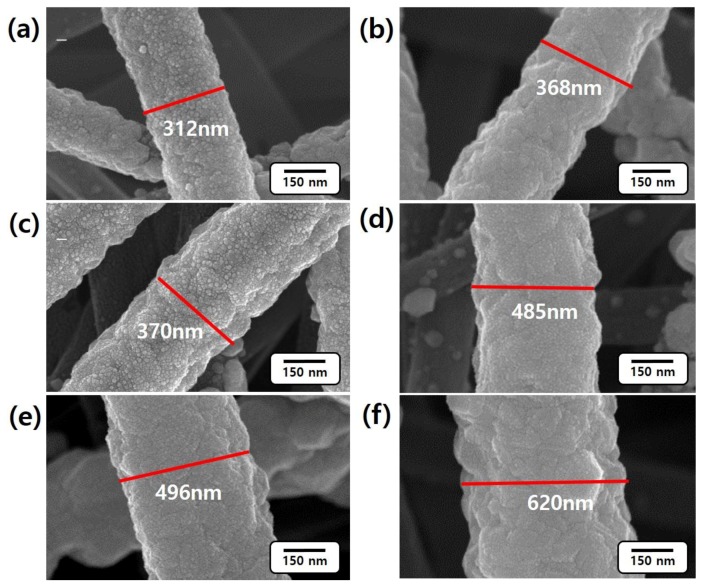
Magnified Pd/CNFs with different deposition time (**a**,**b**) 1 h; (**c**,**d**) 6 h; (**e**,**f**) 12 h is presented. The morphology changes are shown (**a**,**c**,**e**) before and (**b**,**d**,**f**) after hydrogen adsorption.

Hydrogen absorption behavior was investigated by measuring the increase in volume and electric resistance of the samples at different environmental conditions (5%, 10%, 20%, and 50% H_2_). Figure 4 shows FE-SEM images of Pd/CNFs before (Figure 4a,c,e) and after (Figure 4b,d,f) H_2_ adsorption for 1 h. The diameters of 1 h, 6 h, and 12 h Pd/CNFs are increased from 312 nm to 368 nm, from 370 nm to 485 nm and from 496 nm to 620 nm after hydrogen absorption, respectively. The average diameters of 1 h, 6 h, 12 h Pd/CNFs after hydrogen absorption were 389 ± 35 nm, 484 ± 60 nm, and 594 ± 49 nm, which were extracted from the SEM image in Figure S6. The results indicate that H_2_ absorption depends upon the Pd content in the Pd/CNFs. Hydrogen is readily dissociated on Pd surfaces, and the hydrogen atoms then diffuse into the sub-surface layers of the metal and form palladium hydride (PdH*_x_*) [29,30,31]. At the equilibrium state of the hydrogen gas and absorbed atoms, the hydrogen atoms adsorbed on the Pd lattice form Pd-H within the metal lattice [32]. It has been found that hydrogen carries a negative charge in Pd-H and similarly for hydrogen adsorbed on Pd surface [32,33]. As a result, the Pd metal is expanded to increase their volume by adsorbing hydrogen atoms. It is worth noting that the Pd lattice could be expanded from 3.889 Å to 4.025 Å, depending on the amount of hydrogen adsorbed [6]. 

**Figure 5:**
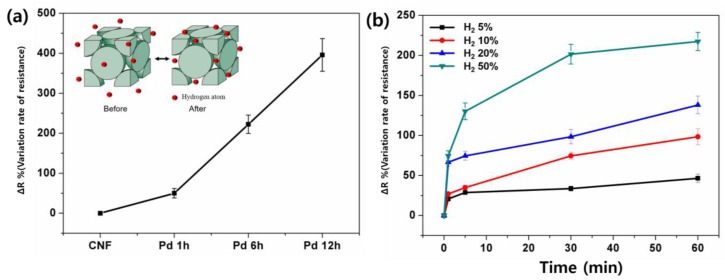
Electro-resistance behavior upon hydrogen adsorption. (**a**) The calculated electric-resistance variation rate (Δ*R*%) with variation in deposition time and (**b**) with different hydrogen concentrations, from 5% (**black**), 10% (**red**), 20% (**blue**), and 50% (**green**).

For further support for the results, comparison experiments of the electro-resistance behavior between hydrogen-absorbed Pd/CNFs and virgin Pd/CNFs for 1 h were conducted (Figure S7). Upon the increase in deposition time, the resistance decreased from 181 Ω to 11 Ω due to the metallic behavior of Pd/CNFs (see the blue column). Moreover, the resistance shows a certain change upon the exposure to hydrogen. The ability to position hydrogen atoms within Pd’s face centered cubic (FCC) lattice structure induces an increment in the resistance of Pd/CNFs. A plausible reaction mechanism during hydrogen exposure has been proposed by Lundstrom et al. [34]; hydrogen molecules are dissolved in the Pd, dissociating molecules into atoms. The variation rate of resistance from Figure S7 was calculated as follows:

∆*R* (%) = (*R*_β_ − *R*_α_)/*R*_α_ × 100 (1)

where ∆*R* is the variation rate of resistance, *R*_α_ is resistance before hydrogen adsorption, and *R*_β_ is resistance after hydrogen adsorption. The variation rate was dramatically increased in 12 h Pd/CNFs to ~400% (~495% in resistance change) compared with that of before hydrogen absorption (Figure 5a). We note that the resistance variation of 12 h Pd/CNFs largely increased compared to other samples. This is likely due to the extraction of Pd in the 12 h sample, which led to the greatest amount of Pd content among the CNFs; a large amount of Pd on a CNF can react with hydrogen molecules easily, resulting in a great transition of conductivity. It may also be assumed that the increase in resistance is caused by a huge amount of adsorption of hydrogen atoms in the Pd lattice. For a sensitivity test, the resistance changes were recorded under a different hydrogen concentration atmosphere. The plot of ∆*R* of 6 h Pd/CNFs in 5%, 10%, 20%, and 50% of hydrogen atmosphere as a function of time is presented in Figure 5b. As expected, the ∆*R* in 50% hydrogen atmosphere shows the highest value from 74% at 1 min to 217% at 60 min. It is worth noting that the Pd/CNF sheet shows rapid response within 1 min in the hydrogen concentration atmosphere. In addition, it shows a higher ∆*R* at a higher hydrogen concentration, showing a ∆*R* that is three times higher in a 50% hydrogen atmosphere compared with that of a 5% hydrogen atmosphere. The results suggest that the fabricated Pd/CNFs can be useful to the rapid detecting hydrogen, which is suitable for utilizing as hydrogen sensing materials. 

## 4. Conclusions

We report here on the successful fabrication of Pd/CNFs for rapid detecting hydrogen molecules at a low hydrogen concentration. The CNFs were fabricated from PAN via stabilization and carbonization processes, severally, and were followed by the chemical deposition of Pd. CNF samples were immersed in a PdCl_2_ solution for 1 h, 6 h, and 12 h, respectively, in an optimized pH condition (pH 5). Prepared samples were highly responsive to electrical resistance changes (~500%) upon exposure to hydrogen. The CNFs have a high surface area for Pd deposition; thus, it could be a good candidate for hydrogen sensing applications. Furthermore, noticeable features of the 1D nanofiber structure have attracted a great deal of attention due to their superior real-time sensitivity and detection with low-power consumption. Fabrication has not been directly related to actual applications for hydrogen sensing utilization yet; however, the Pd/CNFs exhibited a reasonably high responsivity to hydrogen in light of the changes in resistance, indicating that it can be a promising candidate material for hydrogen sensing applications. Furthermore, Pd/CNFs demonstrated a sufficiently high response in electrical resistance changes after adsorbing hydrogen in a low hydrogen environment (5% hydrogen condition). In addition, Pd/CNFs showed rapid response within 1 min in all hydrogen concentration levels, highlighting its potential in the development of hydrogen sensing materials with rapid response, even in low hydrogen concentration conditions. Moreover, the material platform might be useful for various applications, namely, biomedical devices, health care devices, hydrogen batteries, and nuclear power stations for hydrogen sensing.

## Supplementary Materials

The following are available online at http://www.mdpi.com/2079-4991/6/12/226/s1.
