# Supplementary material for: The Chemical Deposition Method for the Decoration of Palladium Particles on Carbon Nanofibers with Rapid Conductivity Changes
Source: Nanomaterials (Basel). 2016 Nov 29;6(12):226. doi: 10.3390/nano6120226 (PMC5302716; doi:10.3390/nano6120226)
Supplement: Supplementary file 1 [file nanomaterials-06-00226-s001.pdf]

Supplementary Materials: The Chemical Deposition Method for the Decoration of Palladium Particles on Carbon Nanofibers with Rapid Conductivity Changes

Hoik Lee, Duy-Nam Phan, Myungwoong Kim, Daewon Sohn, Seong-Geun Oh, Seong Hun Kim and Ick Soo Kim

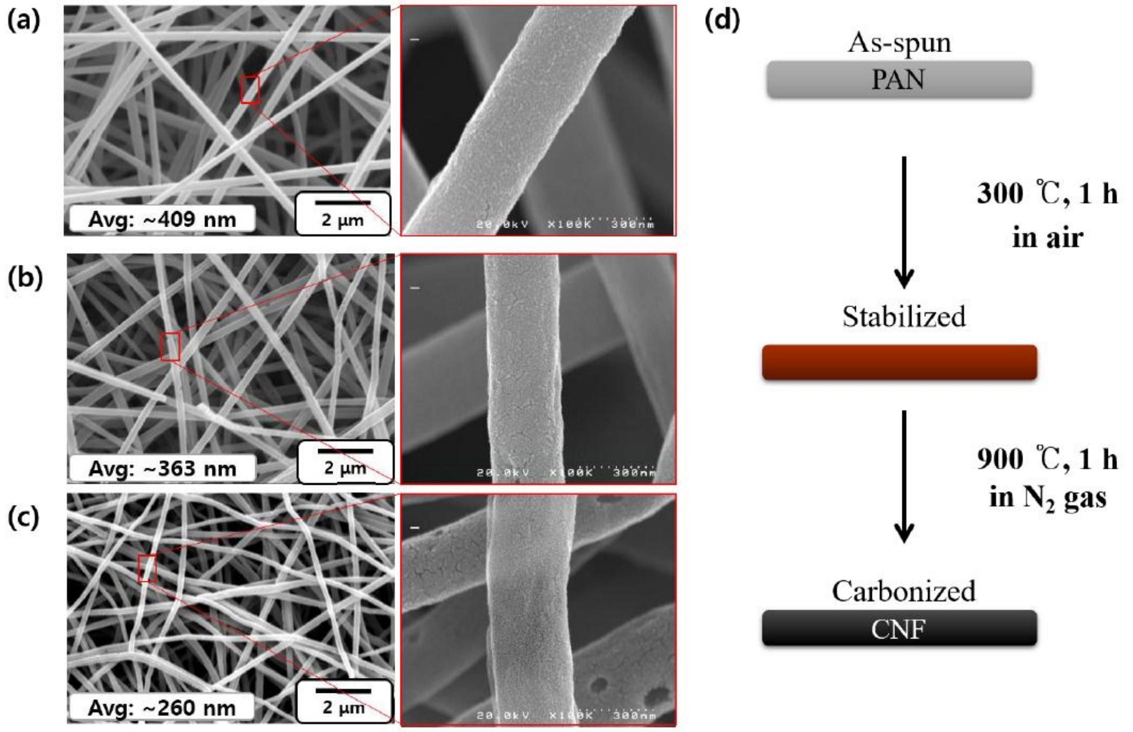

Figure S1. Morphologies of (**a**) PAN; (**b**) ST-NFs; (**c**) CNFs. (**d**) Their schematic illustration via electrospinning, stabilization, and carbonization.

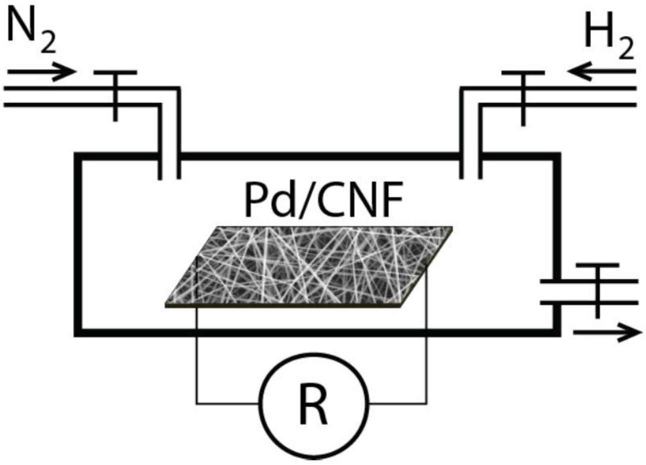

Figure S2. Simple illustration of hydrogen sensing devices.

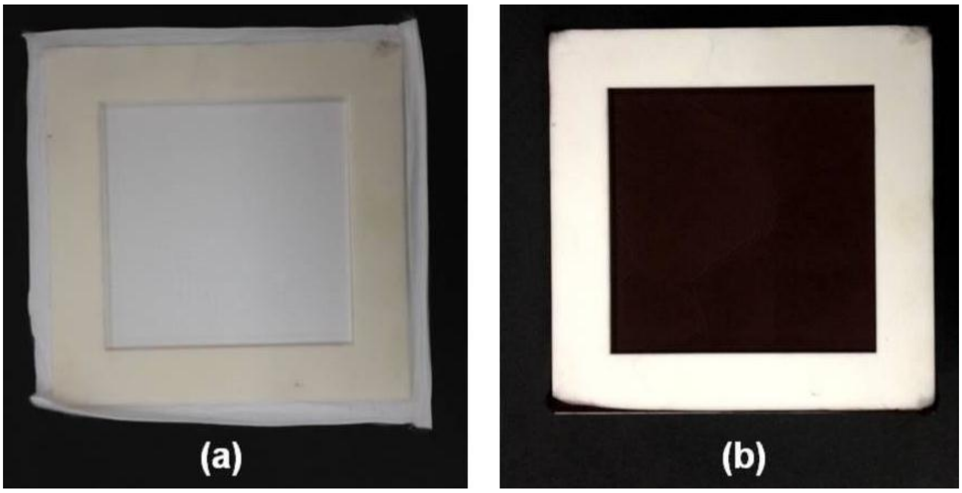

Figure S3. Digital photograph of (**a**) the PAN NF and (**b**) the ST-NF under mechanical stress.

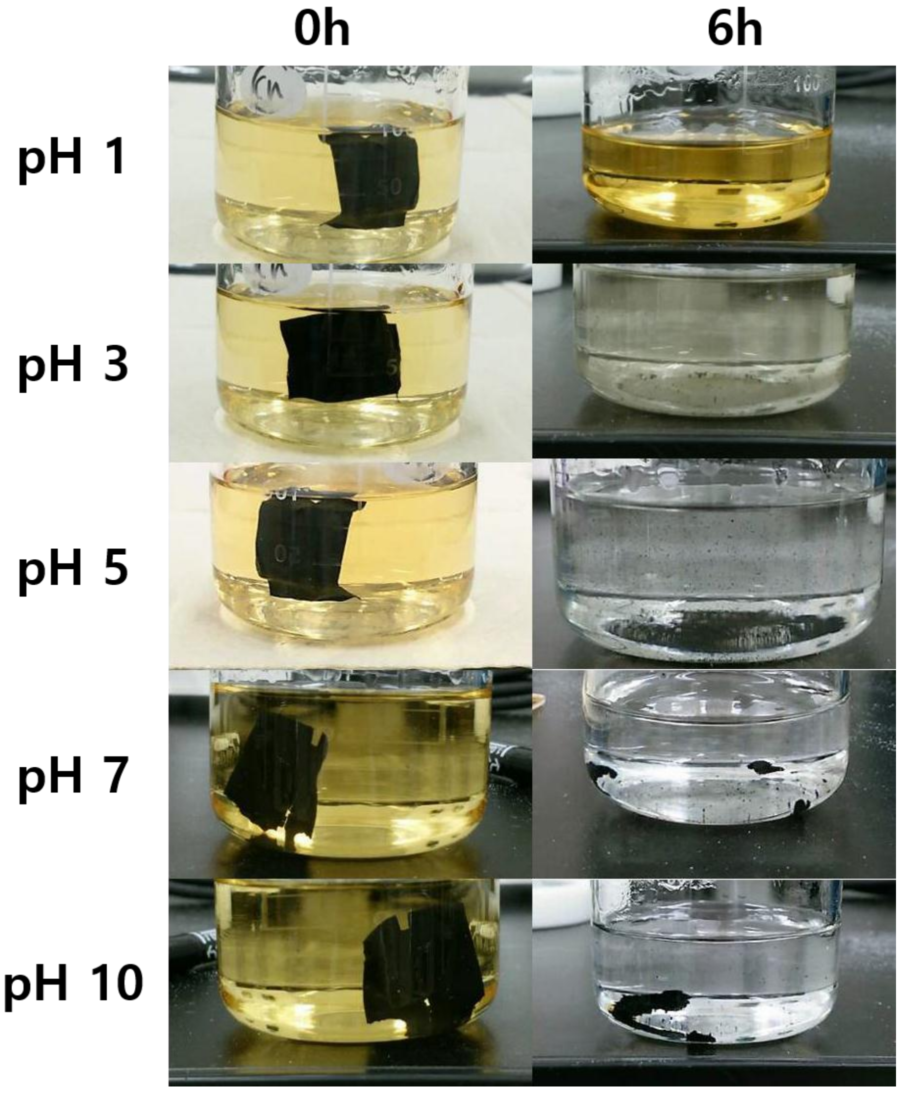

Figure S4. Digital photograph of the Pd deposition process with different pH conditions. (**left**) The initial state of the $PdCl_2$ solution with the CNF, and (**right**) the $PdCl_2$ solution after deposition.

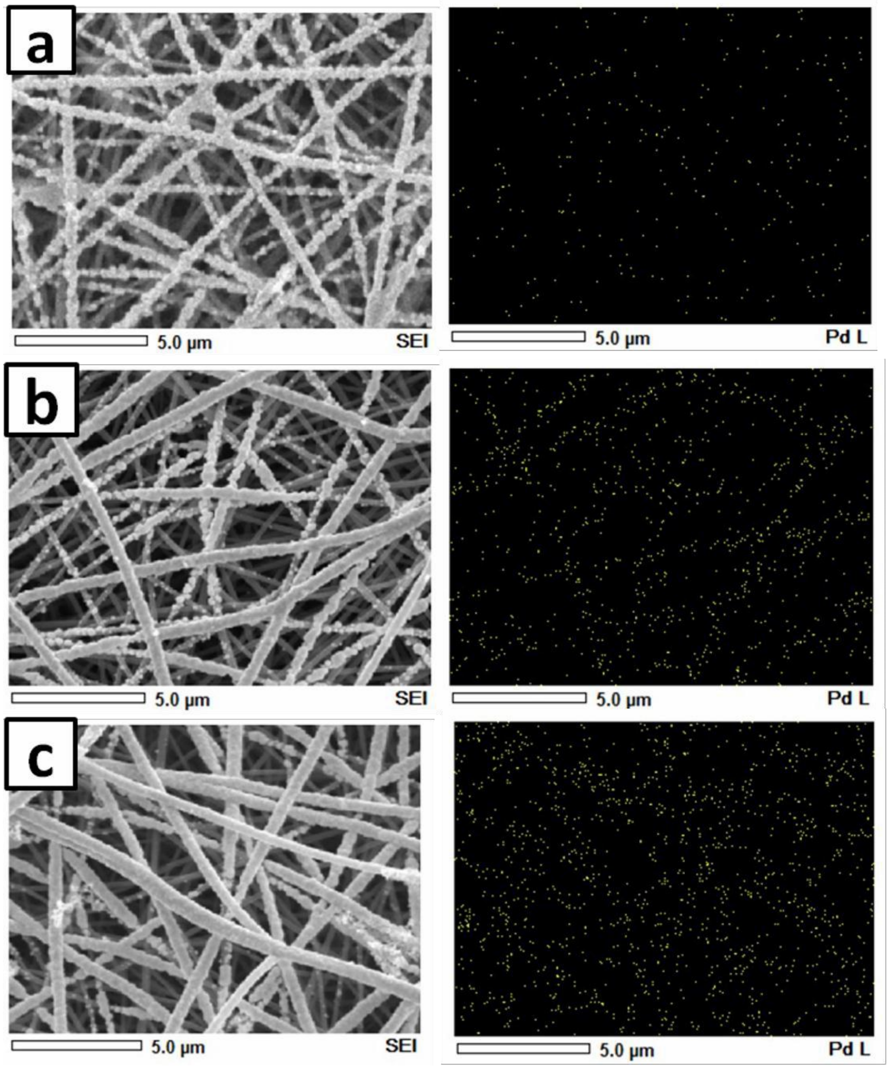

Figure S5. SEM images (**left**) and corresponding to EDX image (**right**) of (**a**) 1 h Pd/CNF; (**b**) 6 h Pd/CNF; and (**c**) 12 h Pd/CNF.

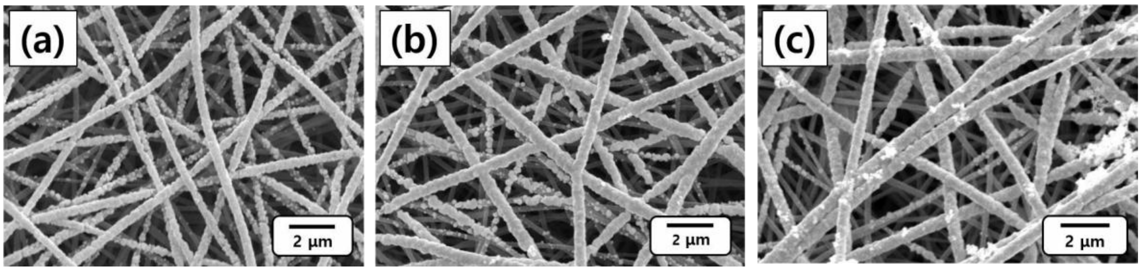

Figure S6. SEM images of (**a**) 1 h; (**b**) 6 h; and (**c**) 12h Pd/CNF after hydrogen absorption.

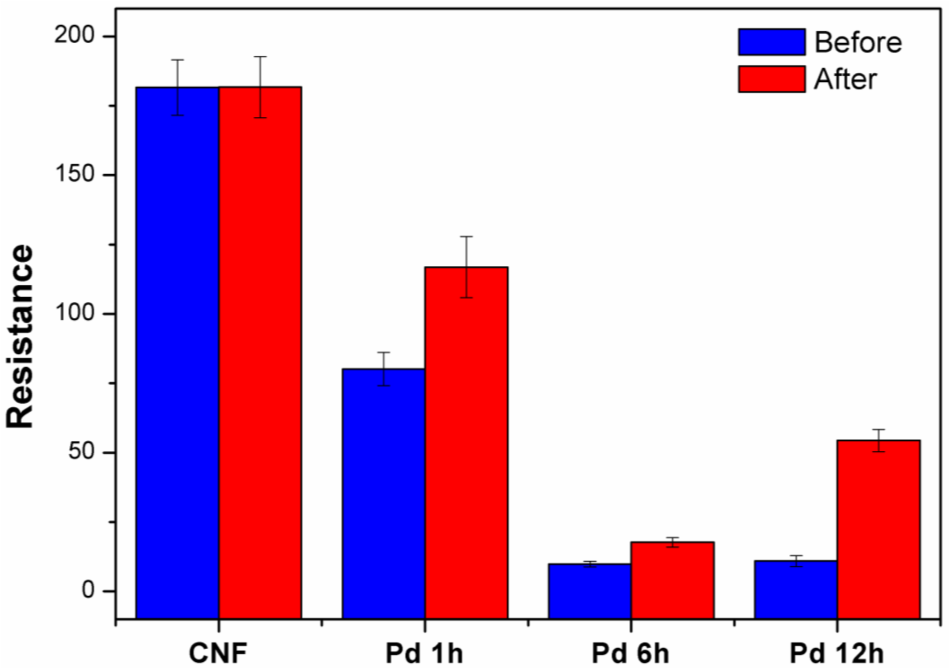

Figure S7. Resistance change before (**blue**) and after (**red**) hydrogen absorption of CNF, 1 h Pd/CNF, 6 h Pd/CNF, 12 h Pd/CNF.

© 2016 by the authors. Submitted for possible open access publication under the terms and conditions of the Creative Commons Attribution (CC-BY) license (http://creativecommons.org/licenses/by/4.0/).
